# The Peptide Network between Tetanus Toxin and Human Proteins Associated with Epilepsy

**DOI:** 10.1155/2014/236309

**Published:** 2014-06-01

**Authors:** Guglielmo Lucchese, Jean Pierre Spinosa, Darja Kanduc

**Affiliations:** ^1^Brain and Language Laboratory, Cluster of Excellence “Languages of Emotions”, Free University of Berlin, 14195 Berlin, Germany; ^2^Faculty of Biology & Medicine, University of Lausanne, CH-1011 Lausanne, Switzerland; ^3^Department of Biosciences, Biotechnologies and Biopharmaceutics, University of Bari, 70125 Bari, Italy

## Abstract

Sequence matching analyses show that *Clostridium tetani* neurotoxin shares numerous pentapeptides (68, including multiple occurrences) with 42 human proteins that, when altered, have been associated with epilepsy. Such a peptide sharing is higher than expected, nonstochastic, and involves tetanus toxin-derived epitopes that have been validated as immunopositive in the human host. Of note, an unexpected high level of peptide matching is found in mitogen-activated protein kinase 10 (MK10), a protein selectively expressed in hippocampal areas. On the whole, the data indicate a potential for cross-reactivity between the neurotoxin and specific epilepsy-associated proteins and may help evaluate the potential risk for epilepsy following immune responses induced by tetanus infection. Moreover, this study may contribute to clarifying the etiopathogenesis of the different types of epilepsy.

## 1. Introduction

The term epilepsy defines a group of disturbances whose only recognized commonality is the paroxysmal synchronous discharging of groups of neurons. Localization and physiological function of the neuronal populations involved determine the clinical picture, so that (1) clinical manifestations can be extremely subtle and the diagnosis can be challenging also in terms of differential definition; (2) epilepsy(ies) can produce extremely multiform clinical pictures with a large degree of overlap [1–3]. Indeed, epileptic syndromes can also be embedded in larger syndromic clinical pictures, that is, West and Lennox-Gastaut syndromes in tuberous sclerosis complex [4, 5]. This clinical diversity has noteworthy nosological implications. Syndromic or disease status of various forms of epilepsy and the terminology used to define them are indeed still matter of debate [7–9]. Likewise, the molecular etiopathogenesis of epilepsies has to be better defined at the molecular level. Although genetic alterations [10–12], inflammation [13], and viral infections [14–16] have been considered and thoroughly studied, nonetheless, the molecular basis and the causal mechanisms of epilepsies are still unclear.

Recently, research on epilepsy has also outlined a neurodevelopmental context [17–21]. Spontaneous recurrent seizures have been observed after induction of* status epilepticus* during the second and third postnatal weeks in rodents, by use of chemoconvulsants such as pilocarpine, kainate, and tetanus toxin (TT) [22]. TT seizures as well as experimental febrile seizures and developmental lithium pilocarpine appear to share a common mechanism for enhancing hippocampal network excitability and promoting epilepsy, possibly through alterations in neurotransmitter receptors or voltage-gated ion channels ([23] and further references therein).

Moreover, numerous reports suggest that immune mechanisms might play a role in processes leading to epileptogenesis [15, 24–32]. In fact, antibodies against neural antigens involved in neurotransmission have been detected in epileptic subjects [33–39], and, remarkably, epilepsy was shown to respond to immunotherapeutic approaches [38, 40, 41]. Finally, population-based cohort studies have documented that microbial infections during pregnancy may be a risk factor for epilepsy in offspring [42–45].

In such a multifaceted scientific-clinical context, here we analyze the peptide commonality between TT, a powerful neurotoxin used in animal models of experimental epilepsy [46–50], and human antigens that have been related to epilepsy, searching for possible immunological link(s) that might contribute to epileptogenesis. Indeed, a massive peptide overlap characterizes microbial and human proteomes [51–54] and gives grounds for questioning whether immune response(s) to microbial infections might potentially result in cross-reactions against neuronal antigens [55–58]. Pathogen versus human immune cross-reactivity might contribute to explaining the association between microbial infections and neurological syndromes [59] and assumes a special significance during pregnancy in light of the consequent possible neurodevelopmental alterations in the fetus and offspring [26, 58].

We report that the tetanus neurotoxin and human epilepsy antigens share an ample pentapeptide platform. The bacterial versus human peptide overlap is not random and, importantly, a search through the Immune Epitope Database (IEDB; http://www.immuneepitope.org/) reveals that the shared pentapeptides are part of TT-derived epitopes. The latter datum is relevant also in light of the role of pentapeptides as minimal functional units in cell biology and immunology [60, 61]. On the whole, the results support the possibility that immune cross-reactions may occur between TT and epilepsy-related proteins.

## 2. Methods

TT protein sequence, UniProtKB/Swiss-Prot accession number: P04958, 1315aa long, from* Clostridium tetani* (NCBI Taxonomic identifier: 212717; further details at http://www.ncbi.nlm.nih.gov/Taxonomy/Browser/wwwtax.cgi) was analyzed for pentapeptide sharing with epilepsy-associated proteins as follows. First, a pentapeptide library was constructed by dissecting the TT primary sequence into pentapeptides offset by one residue, that is, MPITI, PITIN, ITINN, TINNF, INNFR, and so forth. Then, each of the final 1311 pentamers was analyzed for instances of the same match within a library consisting of primary sequences of human proteins that, when altered, have been associated with epilepsy. The number of matches and the human proteins sharing matches were recorded.

Epilepsy-associated proteins were randomly retrieved from UniProtKB Database (http://www.uniprot.org/). An unbiased set of proteins that on whatever basis (i.e., differential regulation, protein modification, or mutation) had been involved in or related to epilepsy was obtained utilizing “epilepsy” and “*Homo sapiens*” as keywords. Only canonical protein sequences were considered. At the time of this study, the keyword-guided search produced a library of 133 human UniProt entries, for a total of 106,022aa. Epilepsy-associated proteins are reported as UniProtKB/Swiss-Prot entry names throughout the paper, unless when discussed in detail. Any pentapeptide occurrence in the set of epilepsy-associated proteins was termed a match.

A set of proteins associated with Down syndrome, a genetic disease in which infectious agents have no role, was retrieved from UniProtKB Database and used as a comparison sample. This set was formed by the following proteins listed according to the aa length, with UniProtKB/Swiss-Prot entries in parentheses: (1) Down syndrome critical region protein 10 (P59022, DSC10), 87aa; (2) Down syndrome critical region protein 8 (Q96T75, DSCR8), 97aa; (3) Down syndrome critical region protein 4 (P56555, DSCR4), 118aa; (4) Down syndrome critical region protein 9 (P59020, DSCR9), 149aa; (5) Down syndrome critical region protein 5 or phosphatidylinositol N-acetylglucosaminyltransferase subunit P (P57054, PIGP), 158aa; (6) Down syndrome critical region protein 6 or protein ripply3 (P57055, DSCR6), 190aa; (7) Down syndrome candidate region 1-like 1 or regulator of calcineurin 2 (Q14206, RCAN2), 197aa; (8) Down syndrome candidate region 1-like protein 2 or regulator of calcineurin 3 (Q9UKA8, RCAN3), 241aa; (9) Down syndrome critical region protein 1 or regulator of calcineurin 1 (P53805, RCAN1), 252aa; (10) Down syndrome critical region protein 2 or proteasome assembly chaperone 1 (O95456, PSMG1), 288aa; and (11) Down syndrome critical region protein 3 (O14972, DSCR3), 297aa.

The Immune Epitope Database (IEDB; http://www.immuneepitope.org/) was used to search for TT-derived B- and/or T-cell epitopes that had been experimentally validated as positive in the human host.

Expected occurrences for pentapeptide sharing between* C. tetani* neurotoxin and human proteins associated with epilepsy were calculated as follows. First, we considered the number of all possible pentapeptides, *N*. Since each residue can be any of 20aa, the number of all possible pentapeptides *N* is given by *N* = 20^5^ = 3.2 × 10^5^. Next, we considered the TT and epilepsy-associated proteins as two sets of pentapeptide size *m* and *n*. That is, *m* is the number of pentapeptides present in the TT protein and *n* is the number of pentapeptides present in the epilepsy-associated protein set. If *X* is the number of times a pentapeptide is selected in the TT protein of size *m* and *Y* is the number of times the same pentapeptide is selected in the epilepsy-associated protein set, then *X* = *m*/*N* and *Y* = *n*/*N*. Assuming that *X* and *Y* are independent, *XY* = *mn*/*N* ^2^. In other words, the expected number of times that one pentapeptide will be selected simultaneously in both TT and epilepsy-related protein set is given by *mn*/*N* ^2^. Neglecting the relative abundance of aa and assuming *m* ≪ *N* and *n* ≪ *N*, we obtain a formula derived by approximation where the total number of occurrences in a second sample *n* (the epilepsy-related protein set) of pentapeptides occurring in the first sample *m* (TT) is given by *mn*/*N* + *m*/2.

## 3. Results and Discussion

### 3.1. Description of the Pentapeptide Sharing between TT and Epilepsy-Associated Proteins

Peptide sharing between TT and human epilepsy-associated proteins was analyzed using (1) the pentapeptide module as a matching probe and (2) a library consisting of 133 epilepsy-related protein sequences retrieved from UniProt (see under Methods).

We used pentapeptides as scanning probes in sequence similarity analyses since a grouping of five aa residues may represent a minimal unit of immune recognition in cellular and humoral responses. Indeed, scientific literature indicates that an optimal peptide length for T-cell epitopes ranges between 9 and 15 residues, with the central 5–7 aa representing the specific immune recognition contacts and the flanking residues determining the binding potential to the MHC molecules [62–66].* De facto*, the HFMPT pentapeptide was reported to be a minimal antigenic determinant for MHC class I-restricted T lymphocytes [65], while the KYVKQ pentapeptide was demonstrated to be a minimal antigenic determinant for CD4(+) T-cell clones [66]; in addition, the IEDB describes numerous pentapeptide epitopes capable of binding MHC molecules (e.g., epitope IEDB IDs: 5740, 7948, 11514, 25472, and 33701) and inducing T-cell proliferation (e.g., epitope IEDB IDs: 815, 40168, 47974, 59947, 107725, 107725, and 110376) (reviewed in [61]). Likewise, humoral immune recognition/reactivity unfolds around short aa motifs ([67–70]; reviewed in [71]). A representative example is a report by Zeng and colleagues [70], according to which the C-terminal pentapeptide (aa sequence: GLRPG) of luteinizing hormone-releasing hormone is a dominant B-cell epitope able to elicit a strong anti-LHRH antibody response and to discriminate between anti-LHRH antibodies present in fertile and nonfertile mice. That is, the pentapeptide GLRPG has immunogenic and antigenic properties and also discriminates antibody specificities associated with reproductive competence.

The analyzed set of 133 human proteins related to epilepsy is listed in Box 1 according to the aa size (i.e., from IR3IP or immediate early response 3-interacting protein 1, 82aa, to GPR98 or monogenic audiogenic seizure susceptibility protein 1 homolog, 6306aa).

Following matching analyses, we found that 42 out of the 133 epilepsy-associated proteins retrieved at random from UniProt database share 58 pentapeptides (68 including multiple occurrences) with the bacterial toxin.  Box 2 lists the epilepsy-related proteins that share pentapeptides with TT and the shared pentapeptides. No TT pentapeptide match was found in the comparison set of proteins associated with Down syndrome.

### 3.2. Nonstochasticity of the Pentapeptide Sharing between TT and Epilepsy-Associated Proteins

The comparative analysis of Boxes 1 and 2 highlights three main points. Firstly, the 68 TT pentapeptide overlap described in Box 2 exceeds the expected value. As detailed under Methods, the expected number of TT pentapeptides that may occur in the epilepsy-related protein set is given by *mn*/*N* + *m*/2, where *m* is the number of pentapeptides contained in TT (1,311), *n* is the number of pentapeptides contained in the epilepsy-related protein set (105,490), and *N* is the number of all possible pentapeptides (20^5^). Developing the equation gives 43 as expected number of pentapeptide matches, whereas the observed value is 68 (see Box 2). That is, the pentapeptide overlap between TT and epilepsy-related proteins is 1.58 times higher when compared to the expected one.

A second point of note is that the distribution of the pentapeptide overlap through the epilepsy-related proteins is unexpected. According to equation described above, pentapeptide sharing between two samples is as a quantity directly proportional to the number of pentapeptides in the analyzed samples; that is, it is proportional to the protein aa size. Actually, 91 epilepsy-related proteins are excluded from the pentapeptide matching with TT, independently of their length. For example, SPTN1, 2472aa (see Box 1), has no bacterial matches, while LRRC1, 524aa, shares 3 pentapeptides with TT (Box 2).

In summary, a comparative analysis of Boxes 1 and 2 highlights that 68 TT pentapeptide matches are allocated in 42 out 133 human proteins that have been related, when altered, to epilepsy, and no relationship appears to exist between pentapeptide sharing and the human protein size. Applying the equation described above to the set of 42 epilepsy-related proteins sharing 68 pentapeptides with TT and amounting to 50,254aa, the expected pentapeptide overlap is equal to 20, so that the observed occurrence value is 3, 4 times higher.

**Figure 1:**
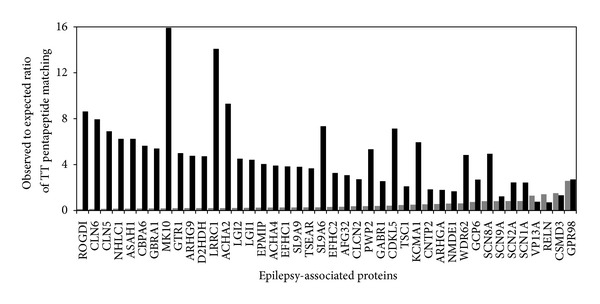
Observed versus expected pentapeptide matching between TT and epilepsy-related proteins. The 42 proteins sharing pentapeptides with TT are allocated along the *x*-axis according to increasing aa length. Gray columns: expected matches calculated according to the formula *mn*/*N* + *m*/2, where *m* is the number of pentapeptides present in the neurotoxin (1,311) and *n* is the number of pentapeptides present in the epilepsy-associated protein (see Methods). For example, in the case of IR3IP protein, 82aa, the possible pentapeptide overlap is equal to 1,311 × 78/3,200,000 + 1,311/2. Black columns: observed to expected ratio of the pentapeptide matching. Observed matching values from Box 2.

Finally, a third* punctum saliens* is that nonrandomness characterizes also the distribution of the TT pentapeptides among the 42 epilepsy-associated proteins.  Box 2 shows that a few TT pentapeptides are repeated in the 42 epilepsy-associated protein set. Indeed, TT pentapeptides EIIPS, SLSIG, and FCKAL recur twice, and TT pentapeptides FGGQD, KEIEK, and TFLRD occur three times (Box 2; see pentapeptides underlined).  Box 2 also shows that MK10 (mitogen-activated protein kinase 10; 464aa); CDKL5 (cyclin-dependent kinase-like 5; 1030aa); and KCMA1 (calcium-activated potassium channel subunit alpha-1; 1236aa) share two sequentially overlapping pentapeptides with TT, that is, share the hexapeptides SVDDAL, KNSFSE, and PKEIEK, respectively. The nonrandom TT pentapeptide sharing clearly emerges from Figure 1, where expected and observed occurrence values are graphically compared.

It can be seen that, in conflict with the theoretical trend of the TT pentapeptide matching as a function of epilepsy-related protein length (Figure 1, columns in gray), the observed to expected ratio of pentapeptide matching shows no relationship with the human protein length (Figure 1, columns in black). For example, contrary to mathematical expectations, MK10 (464aa long) has three pentapeptide matches, whereas VP13A (3174aa long) has one match (see Box 2 and Figure 1).

### 3.3. Immunologic Potential of the Pentapeptide Sharing between TT and Epilepsy-Associated Proteins

Having defined the TT versus epilepsy-associated proteins pentapeptide overlap, it was next tested whether such a sharing has an immunologic potential. To this aim we used IEDB, a database that describes B- and T-cell epitopes for humans, nonhuman primates, rodents, and other animal species, and searched for TT-derived epitopes that had been validated as immunopositive in humans. At the time of the search, we obtained a list of 517 TT-derived epitopes. The pentapeptides common to epilepsy-associated proteins and TT (see Box 2, sequences in italic) were used as probes to scan the 517 TT-derived epitope set in order to define potential cross-reactive peptide sequences. Results are reported in Table 1.

In essence, Table 1 shows that all of the 58 pentapeptides common to the 42 epilepsy-associated proteins and TT (Box 2, peptide sequences in parentheses and in italic) are present in 116 TT-derived epitopes that had been established to be immunopositive in humans. This datum indicates a potential vulnerability of the 42 epilepsy-associated proteins to cross-reactions following anti-TT immune responses. Moreover, many TT-derived epitopes share fragments with distinct epilepsy-related proteins and are of particular significance to a multiple cross-reactivity risk, since, for example, an immune response targeting the TT epitope fnnftVSFWLRVPKVsahle (see Table 1, IEDB ID 17207, with shared fragments in capital letter) has the potential to cross-react with the following three crucial proteins related to different forms of epilepsy:

- GBRA1 or gamma-aminobutyric acid receptor subunit alpha-1, the major inhibitory neurotransmitter in the vertebrate brain that mediates neuronal inhibition by binding to the GABA/benzodiazepine receptor and opening an integral chloride channel [72],
- SCN8A or voltage-gated sodium channel subunit alpha Nav1.6, a protein that mediates the voltage-dependent sodium ion permeability of excitable membranes [73],
- EFHC1 or myoclonin-1, a protein that may enhance calcium influx through CACNA1E and stimulate programmed cell death [74].

Such a multiple cross-reactivity potential is shown also by other TT-derived epitopes, eg, epitopes IEDB IDs 30436, 48049, 113407, and so forth.

Also, it seems important to highlight that MK10 (mitogen-activated protein kinase 10, also known as stress-activated protein kinase JNK3 or p493F12 kinase), a protein that shows the highest unexpected level of pentapeptide overlap to TT (Figure 1) and also has a high immunologic potential as illustrated in Table 1 (i.e., MK10 pentapeptide(s) are present in 7 TT-derived epitopes), is selectively expressed in a subpopulation of pyramidal neurons in the CA1, CA4, and subiculum regions of the hippocampus, and layers 3 and 5 of the neocortex [75]. That is, there is a potential cross-reactivity risk specifically allocated in brain areas directly linked to epileptogenesis [76, 77].

## 4. Conclusions

This study describes a vast pentapeptide commonality between TT-derived epitopes and epilepsy-associated proteins. This peptide sharing acquires a relevant pathologic potential in light of the fact that pentapeptide modules have the capacity of inducing immune response(s) and are main players in immune recognition [61–71]. Immunologically, two sequences that share a pentapeptide are potentially subject to a cross-reaction [60].

In the disease model examined here, that is, tetanus infection and epilepsy, the ample cross-reactivity platform between TT-derived epitopes and human epilepsy-associated antigens supports the hypothesis of an immune involvement in epilepsy. As a matter of fact, all the 42 epilepsy-related proteins listed in Box 2 are potential targets of cross-reactions (see Table 1). Qualitatively, the peptide overlap occurs in human proteins canonically associated with epilepsy such as gamma-aminobutyric acid receptor subunit alpha-1 (GBRA1), gamma-aminobutyric acid type B receptor subunit 1 (GABR1), sodium channel protein subunits (SCN1A, SCN2A. SCN8A, and SCN9A), and calcium-activated potassium channel subunit alpha-1 (KCMA1) (Table 1). Obviously, an immune attack against such epilepsy-associated proteins may cause alterations to neural structures and functions, especially when the neurodevelopmental intrauterine phase is considered. Being of nonsecondary importance, the nonstochastic character of the peptide overlap between TT and epilepsy-associated proteins (Figure 1) indicates that the potential cross-reactivity extent (and the associated risk of developing epilepsy and neurodevelopmental disorders) will increase with the number of anti-TT immune stimulations.

An additional relevant point is the “antigenic patchwork” shown in Table 1. Indeed, the potential peptide crossreactome involved in different extent and in different combinations of 42 epilepsy-associated proteins might help understand the complex neurobiological network that, once hit and perturbed, may underlie different epileptic forms [1–9].* Also*, it has to be noted that Table 1 includes proteins such as CNTP2 or contactin-associated protein-like 2, RELN or reelin, and TSC1 or tuberous sclerosis 1 protein, which are also landmark antigens for autism and the associated impairment in communication/language skills and behaviors [78–81]. Hence, Table 1 may provide a mechanistic framework to allocate the occurrence of epilepsy, intellectual disability, and autism spectrum disorder in patients with tuberous sclerosis complex. Likewise, data from Table 1 might contribute to answering a critical question in neuropsychopathology, that is, the coexistence of patients with combined schizophrenia and epilepsy [82–85]. Indeed, Table 1 substantiates the hypothesis according to which the thread joining epilepsy and schizophrenia may reside in neurodevelopmental molecules such as leucine-rich glioma inactivated (LGI) proteins and GPR98, a G protein-coupled receptor, originally known as VLGR1 or very large G protein-coupled receptor [86].* De facto*, Table 1 shows that fragments from LGI1, LGI2, and GPR98 are present in 1, 7, and 18 TT-derived epitopes, respectively. In other words, the potential cross-reactivity targeting LGI1, LGI2, and GPR98 following an anti-TT response is high.

Given the caveat that peptide immunoreactivity is influenced by numerous factors, for example, binding affinity [87], cripticity (i.e., determinants embedded in membrane structures do not induce immune responses under physiological conditions) [88], and posttranslational modifications (i.e., citrullination) [89], the present data might contribute to further our understanding of epilepsies. In particular, data from Table 1 might represent a peptide platform to be tested in antibody binding assays using sera from epileptic subjects. Accompanied by parallel immunoassays based on the utilization of epilepsy-related proteins as antigens, such an approach might not only validate the TT-epilepsy link proposed in this study, but also lead to a definition at the molecular level of the repeatedly advanced association between antibodies and epilepsy [33–41]. Moreover, of not less importance, immunoassay validation could also represent a prelude to specific therapies based on peptide modules able to block epileptogenic anti-TT autoantibodies [38–41, 90]. Immunological research in this direction has been programmed in our lab.

## Figures and Tables

**Box 1:**
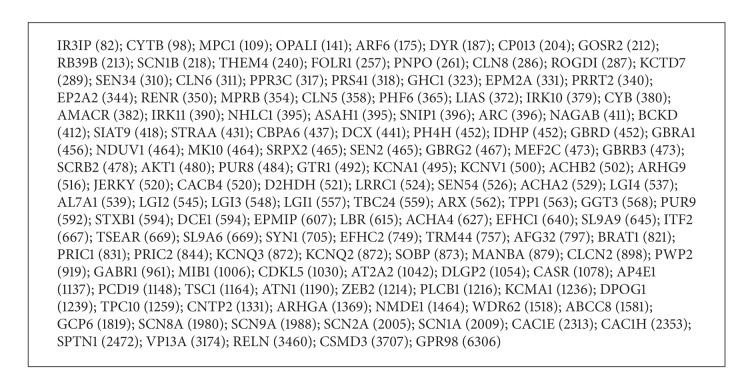
List of the 133 epilepsy-associated proteins analyzed for TT pentapeptide sharing. Proteins were randomly retrieved from UniProtKB (http://www.uniprot.org/) as described under Methods. Proteins are indicated by UniProtKB/Swiss-Prot entry names, and listed according to increasing aa length reported in parentheses.

**Box 2:**
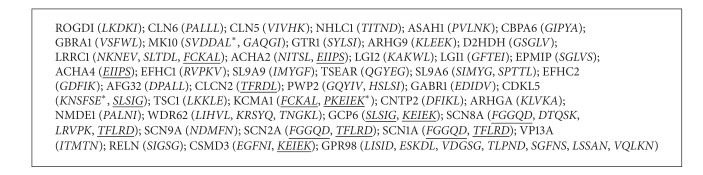
Peptide sharing between TT and epilepsy-associated proteins. Proteins reported by UniProtKB/Swiss-Prot entry names and listed according to the aa length. Pentapeptides shared with TT are italic in parentheses. Pentapeptides present more than once in the epilepsy antigen set are underlined. Sharing of two consecutively overlapped pentapeptides (i.e., a hexapeptide) is indicated by an asterisk.

**Table 1:** Pentapeptide sharing between TT-derived epitopes and human epilepsy-associated proteins.

IEDB ID^1^	TT-derived epitope^2,3^	Immune context	Epilepsy-associated proteins^4^
1270	afcpeyvptfdnvieNITSL	HLA-Class II, allele undetermined	ACHA2
1389	afrnVDGSGLVSklig	HLA-Class II, allele undetermined	GPR98 D2HDH EPMIP
1501	agevrqiTFRDLpdkfnayl	HLA-Class II, allele undetermined	CLCN2
1929	aihlvnnesseVIVHKamdi	HLA-DRB1*04:01	CLN5
2219	akkqllefDTQSKnilmqyi	HLA-Class II, allele undetermined	SCN8A
3156	amltnliifgpgPVLNKNEV	HLA-Class II, allele undetermined	ASAH1 LRRC1
3418	anskfigiteLKKLEskink	HLA-DRB1*11:01	TSC1
3832	apsyTNGKLniyyrrlyngl	HLA-DRB5*01:01, HLA-DRB1*13:01	WDR62
7603	danLISIDikndlyektl	HLA-DRB1*03:01	GPR98
8734	dinndiisdiSGFNSsvity	HLA-DRB1*01:01	GPR98
8778	diSGFNSsvitypdaqlvpg	HLA-DRB1*15:01	GPR98
/8903	dkisdvstivpyigPALNIv	HLA-DPB1*04:01, HLA-DRB1*15:01	NMDE1
9297	dltfiaeKNSFSEepfqdei	HLA-DRB1*01:01, HLA-DRB1*04:01	CDKL5
9595	DPALLLmheLIHVLhglyg	B-cell HLA-DR2; HLA-Class II, allele undetermined	AFG32 CLN6 WDR62
9595	drLSSANlyingvlmgsaei	B-cell HLA-DR2; HLA-Class II, allele undetermined	GPR98
10472	DTQSKnilqyikanskfigiteLKKLEski	HLA-Class II, allele undetermined	SCN8A TSC1
11980	efDTQSKnilmqyikanskfigitel	B-cell	SCN8A
13095	eLIHVLhglygmqvss	B-cell HLA-DR2; HLA-Class I, allele undetermined	WDR62
13125	eLKKLEskinkvfstpipfs	HLA-Class II, allele undetermined	TSC1
13813	eqdpsgattksamltnliifgpgPVLNKNEV	HLA-Class II, allele undetermined	ASAH1 LRRC1
15087	eysiessmkkHSLSIGSGwsvsl	B-cell	PWP2 GCP6 CDKL5 RELN
15411	fdkdsnGQYIVnedkfqily	HLA-Class II, allele undetermined	PWP2
16155	fiaeKNSFSEepfqdeivsyntk	B-cell	CDKL5
17134	fnaylankwvfiTITNDrls	HLA-Class II, allele undetermined	NHLC1
17205	fnnftVSFWLRVPK	HLA-Class II, allele undetermined	GBRA1 SCN8A
17206	fnnftVSFWLRVPKVsahle	HLA-DR3	GBRA1 SCN8A EFHC1
17207	fnnftVSFWLRVPKVsashle	HLA-DRB1*11:01, HLA-DR, HLA-DR1, HLA-DR5, HLA-DR7, HLA-DR11, HLA-DPw4, HLA-Class II, allele undetermined	GBRA1 SCN8A EFHC1
17208	fnnftVSFWLRVPKVsashleqy	HLA-DRB1*01:01, HLA-DRB1*04:01, HLA-HLA-DRB1*07:01, HLA-DRB1*11:01	GBRA1 SCN8A EFHC1
17487	fqilynSIMYGFTEIelgkk	HLA-Class II, allele undetermined	SL9A6 SL9A9 LGI1
18217	fvksGDFIKLyvsynnnehivgy	B-cell	EFHC2 CNTP2
18356	fwLRVPKVsashleqygtne	HLA-DRB1*11:01	SCN8A EFHC1
19469	gevrqiTFRDLpdkfnaylankw	B-cell	CLCN2
21599	gpdkeqiadeinnlknKLEEKan	B-cell	ARHG9
22769	gtneysiissmkkHSLSIGS	DQB1*06:02, DRB5*01:01	PWP2 GCP6 CDKL5
24238	hLKDKIlgcdwyfvptdegwtnd	HLA-Class II, allele undetermined	ROGDI
25597	idkisdvstivpyigPALNI	HLA-Class II, allele undetermined	NMDE1
25666	idsfvksGDFIKLyvsynnn	HLA-DRB1*15:01	EFHC2 CNTP2
26808	ikiknedltfiaeKNSFSEe	HLA-Class II, allele undetermined	CDKL5
27639	ingkaihlvnnesseVIVHK	HLA-Class II, allele undetermined	CLN5
29241	ivdynlqskiTLPNDrttpv	HLA-Class II, allele undetermined	GPR98
29331	ivkQGYEGnfig	HLA-Class II, allele undetermined	TSEAR
29407	ivpyigPALNIv	HLA-Class II, allele undetermined	NMDE1
29408	ivpyigPALNIvkQGYEGnf	HLA-DRB1*15:01	NMDE1 TSEAR
29843	KAKWLgtvntqfqKRSYQ	HLA-Class II, allele undetermined	LGI2 WDR62
29891	kamdieyNDMFNnftVSFWLrvp	B-cell	SCN9A GBRA1
30269	kdVQLKNitdymyltnapsy	HLA-DRB1*01:01, HLA-DRB1*04:01	GPR98
30436	KEIEKlytSYLSITFLRDpwgnp	B-cell	CSMD3 KCMA1 GCP6 GTR1 SCN1A SCN2A SCN8A CLCN2
30572	keqiadeinnlknKLEEKan	HLA-Class II, allele undetermined	ARHG9
32521	knitdymyltnapsyTNGKL	HLA-Class II, allele undetermined	WDR62
32546	knldcwvdneEDIDVilkkstil	B-cell	GABR1
33527	kstilnldinndiisdiSGFNSs	B-cell	GPR98
34301	kwievyKLVKAKWLgtvntq	HLA-DRB1*01:01	ARHGA LGI2
34887	lankwvfiTITNDrLSSANlyin	B-cell	NHLC1 GPR98
35058	lcikiknedltfiaeKNSFS	HLA-DRB1*04:01	CDKL5
35566	lekryekwievyKLVKAKWL	HLA-Class II, allele undetermined	ARHGA LGI2
35993	lftFGGQDanLISIDikndl	HLA-Class II, allele undetermined	SCN1A SCN2A SCN8A GPR98
36667	lipvassskdVQLKNitdym	HLA-DRB1*11:01	GPR98
38977	lqrITMTNSVDDALinstki	HLA-Class II, allele undetermined	VP13A MK10
40770	lygmqvsshEIIPSkqeiym	HLA-Class II, allele undetermined	ACHA2 ACHA4
41527	mfnnftVSFWLRVPKVsash	HLA-DRB1*11:01	GBRA1 SCN8A EFHC1
42847	mtnSVDDALinstkiysyfp	HLA-DRB1*11:01	MK10
43280	napsyTNGKLniyyrrlynglkf	B-cell	WDR62
43519	ndrLSSANlyingvlmgsae	HLA-Class II, allele undetermined	GPR98
43591	neEDIDVilkkstilnldin	HLA-Class II, allele undetermined	GABR1
43939	nftVSFWLRVPK	HLA-Class II, allele undetermined	GBRA1 SCN8A
43940	nftVSFWLRVPKVsashle	HLA-DRB1*11:01	GBRA1 SCN8A EFHC1
44007	ngkaihlvnnesseVIVHKamdi	B-cell	CLN5
44396	nivkQGYEGnfi	HLA-Class II, allele undetermined	TSEAR
44200	niddntiyqylyaqkSPTTL	HLA-DRB1*01:01	SL9A6
44383	NITSLtigkskyfqDPALLL	HLA-ClassII, allele undetermined	ACHA2 AFG32 CLN6
44557	NKNEVrgivlrvdnknyfpc	HLA-Class II, allele undetermined	LRRC1
44667	nldinndiisdiSGFNSsvi	HLA-Class II, allele undetermined	GPR98
45102	nnftVSFWLRVPKVsashle	HLA-Class II, allele undetermined	GBRA1 SCN8A EFHC1
46136	ntiyqylyaqkSPTTLqrit	HLA-Class II, allele undetermined	SL9A6
46853	PALLLmheLIHVLhglygmq	HLA-Class II, allele undetermined	CLN6 WDR62
46855	PALNIvkQGYEGnfigalet	HLA-Class II, allele undetermined	NMDE1 TSEAR
48049	PKEIEKlytSYLSITFLRDf	HLA-Class II, allele undetermined	GCP6 CSMD3 KCMA1 GTR1 SCN1A SCN2A SCN8A CLCN2
48697	pnrdiliasnwyfnhLKDKIlgc	B-cell	ROGDI
49984	pvtkGIPYApeyksnaastteih	B-cell	CBPA6
51254	qkSPTTLqrITMTNSVDDALIns	B-cell	SL9A6 VP13A MK10
56528	ryekwievyKLVKAKWLgtvntq	B-cell	ARHGA LGI2
57935	sfvksGDFIKLyvsynnneh	HLA-ClassII, allele undetermined	EFHC2 CNTP2
57947	sfwLRVPKVsashle	HLA-DR5, HLA-DRB1*11:01	SCN8A EFHC1
58527	SIGSGwsvslkgnnliwtlk	HLA-DRB1*03:01	RELN
59500	SLTDLggelcikikn	HLA-Class II, allele undetermined	LRRC1
61214	ssmkkHSLSIGSGwsvslkg	HLA-Class II, allele undetermined	PWP2 GCP6 CDKL5 RELN
61354	ssskdVQLKNitdymyltnapsy	B-cell	GPR98
62073	SVDDALinstkiysyfpsviskvnqGAQGIl	HLA-Class II, allele undetermined	MK10
63277	tdymyltnapsyTNGKLniy	HLA-DRB1*01:01, HLA-DRB1*04:01	WDR62
63450	teLKKLEskinkvfstpipf	HLA-DRB1*07:01	TSC1
64514	tiyndtEGFNIESKDLksey	HLA-Class II, allele undetermined	CSMD3 GPR98
65324	TNGKLniyyrrlynglkfii	HLA-Class II, allele undetermined	WDR62
67104	tvntqfqKRSYQmyrsletqvda	B-cell	WDR62
67147	tVSFWLRVPKVsa	HLA-DRB1*11:01, HLA-DRB1*11:04	GBRA1 SCN8A EFHC1
67148	tVSFWLRVPKVsashle	HLA-DRB1*11:01	GBRA1 SCN8A EFHC1
68104	vdynlqskiTLPNDrttpvt	HLA-DQB1*06:02	GPR98
69149	VIVHKamdieyNDMFNnftv	HLA-Class II, allele undetermined	CLN5 SCN9A
69180	vKAKWLgtvntqfqKRSYQm	HLA-DQB1*06:02	LGI2 WDR62
70202	vntqfqKRSYQmyrsleyqv	HLA-DRB1*07:01	WDR62
70165	vnqGAQGIlflqwvrdiidd	HLA-Class II, allele undetermined	MK10
70166	vnqGAQGIlflqwvrdiiddftn	B-cell	MK10
70514	vpyigPALNIvk	HLA-Class II, allele undetermined	NMDE1
70982	vsidkfriFCKALnpk	HLA-DRB1*11:01	LRRC1 SCN8A KCMA1
71155	vstivpyigPALNI	HLA-DR, HLA-DR1, HLA-A*02:01	NMDE1
71156	vstivpyigPALNIvkQGYEGnf	B-cell	NMDE1 TSEAR
72784	wLRVPKVsashleqygtneysie	B-cell	SCN8A EFHC1
76411	yvsidkfriFCKALnPKEIE	HLA-Class II, allele undetermined	LRRC1 KCMA1^5^
76537	yylipvassskdVQLKNitd	HLA-Class II, allele undetermined	GPR98
79808	eLIHVLhglygmq	HLA-DRA*01:01, HLA-DRB1*01:01	WDR62 KCMA1
79816	evyKLVKAKWLgt	HLA-DRA*01:01, HLA-DRB1*01:01	ARHGA LGI2
113407	fnnftVSFWLRVPKVsas	HLA-DR11	GBRA1 SCN8A EFHC1
167585	glygmqvsshEIIPSkqeiy	HLA-DRB1*12:01	ACHA2 ACHA4
167613	kvnqGAQGIlflqwvrdiid	HLA-DRB1*12:01	MK10
167626	nLISIDikndlyektlndyk	HLA-DRB1*12:01	GPR98
167666	shEIIPSkqeiymqhtypis	HLA-DRB1*12:01	ACHA2 ACHA4

^1^One hundred and sixteen linear TT-derived epitopes that had been found to be immunopositive in the human host were analyzed. Epitope number refers to IEDB ID. Further details and references are reported in the Immune Epitope Database (IEDB; http://www.immuneepitope.org/.

^2^Aa sequences given in one-letter code.

^3^Peptide fragments shared with epilepsy-associated proteins in capital.

^4^Epilepsy-associated proteins reported as UniProt/Swiss-prot entries. For details and references, see http://www.uniprot.org/.

^5^TT-derived epitope ID 76411 shares both pentapeptides FCKAL and PKEIE with human KCMA1 (or calcium-activated potassium channel subunit alpha-1).
